# Portable low-flow nasal oxygen versus ambient air in patients with chronic lung disease and exercise-induced hypoxaemia: a randomised controlled crossover non-inferiority trial

**DOI:** 10.1136/bmjresp-2025-004048

**Published:** 2026-09-21

**Authors:** Mona Lichtblau, Carmen Wick, Anna Titz, Julian Müller, Simon Raphael Schneider, Dinah Hertig, Zoe Bousraou, Alessandro Vella, Michael Furian, Malcolm Kohler, Esther Irene Schwarz, Silvia Ulrich

**Affiliations:** 1 Department of Pulmonology, University Hospital Zurich, Zürich, Switzerland; 2 Faculty of Medicine, University of Zurich, Zürich, Switzerland

**Keywords:** Pulmonary Disease, Chronic Obstructive, Ambulatory Oxygen Therapy, Interstitial Fibrosis

## Abstract

**Background:**

Exercise-induced desaturation is a common symptom in chronic lung disease (CLD) and is associated with functional limitation and increased mortality. Although portable low-flow supplemental oxygen therapy (SOT) is commonly prescribed for exercise-induced hypoxaemia, evidence for SOT to improve performance is inconsistent.

**Method:**

This pragmatic, randomised, controlled, non-inferiority crossover trial enrolled patients with chronic obstructive pulmonary and interstitial lung disease who exhibited exercise-induced oxygen desaturation of >3%. Each patient performed two 6-minute walk distance (6MWD) tests in randomised order: one breathing ambient air and one using low-flow SOT delivered by a portable backpack concentrator (1–2 L/min). The primary outcome was 6MWD, secondary outcomes included perceived exertion and preference of the interventions. A predefined non-inferiority margin of 35 m was used.

**Results:**

Forty patients were enrolled (mean age 66±13 years; 14 women; 20 with chronic obstructive pulmonary disease (COPD), 20 with interstitial lung disease (ILD)). The mean difference in 6MWD between ambient air and SOT was 0 m (95% CI −14 to 13 m; p<0.001 for non-inferiority). Secondary outcomes demonstrated significantly lower dyspnoea scores with SOT both overall and within COPD/ILD subgroups. Patient preferences were split, with 49% favouring ambient air and 51% preferring SOT. A clinically meaningful improvement in 6MWD of >35 m was found in five patients.

**Conclusion:**

In patients with CLD and exercise-induced desaturation, ambient air was non-inferior to pulsed low-dose SOT by portable concentrator in 6MWD, but SOT improved dyspnoea. These findings highlight the need for more effective, patient-acceptable oxygen delivery systems and further research to identify responders to SOT.

**Trial registration number:**

NCT06385301.

**WHAT IS ALREADY KNOWN ON THIS TOPIC**

- Portable low-flow supplemental oxygen therapy (SOT) is commonly prescribed for exercise-induced hypoxaemia.
- Evidence for portable low-flow SOT ability to improve exercise performance is inconsistent.
- Notably, most studies demonstrating performance benefits have used higher oxygen flow rates than those typically provided by portable low-flow systems.

**WHAT THIS STUDY ADDS**

- In patients with chronic lung disease (chronic obstructive pulmonary disease, interstitial lung disease) and exercise-induced oxygen desaturation, walking in ambient air was non-inferior to pulsed low-dose SOT administered via nasal cannula from a commonly prescribed, portable backpack concentrator.

**HOW THIS STUDY MIGHT AFFECT RESEARCH, PRACTICE OR POLICY**

- The used flow of currently available mobile concentrators might be too low for an impact on exercise capacity.
- Baseline 6-minute walk distance appears to influence responsiveness to SOT, with lower distances in ambient air being associated with a greater likelihood of improvement.

## Background

 Patients with chronic lung diseases (CLD) including chronic obstructive pulmonary disease (COPD) or interstitial lung disease (ILD) commonly experience exercise-induced oxygen desaturation with the frequency increasing as the severity of the CLD progresses.^1–3^ The general prevalence of exercise-induced desaturation in those populations is reported to be between 50% and 60%.^2–4^ Exercise-induced desaturation is more than a pathophysiological observation. It is strongly associated with functional limitation^2 3^ and higher mortality.^5^ In COPD, desaturation during exertion results from ventilation-perfusion mismatch,^6 7^ reduced diffusion capacity, increased physiological dead space and mechanical restriction due to hyperinflation,^8^ leading to severe exercise limitation, disability and reduced quality of life.^9 10^ Reduced physical activity is in turn linked to a higher rate of hospitalisations and mortality,^11–14^ while better preservation of physical activity contributes to longer survival.^14^ Similarly, in ILD, exertional desaturation during the 6-minute walk test (6MWT) is known to be associated with increased mortality, especially in idiopathic pulmonary fibrosis.^15^

As physical activity is crucial for functional autonomy and prognosis, strategies to improve exercise capacity are of high clinical relevance. Despite paucity of evidence, supplemental oxygen therapy (SOT) is commonly prescribed to patients with exercise-induced desaturation, aiming to enhance survival and alleviate symptoms. German and Swiss pulmonary societies recommend ambulatory SOT for exercise-induced hypoxaemia.^16 17^

High-dose SOT has shown short-term benefits on exercise performance in COPD, pulmonary hypertension and heart disease.^18–20^ However, the evidence for the commonly used low-dose nasal oxygen is less robust. In COPD, results are conflicting^21–23^ and in ILD, several studies showed short-term improvements in exercise performance,^24–26^ but effects on dyspnoea and quality of life remain inconsistent.^24 26^ A systematic review showed an immediate benefit of SOT in idiopathic pulmonary fibrosis (mean difference in 6-minute walk distance (6MWD) 48 m), but they did not make a distinction between short-term and long-term oxygen therapy. Moreover, many studies have applied higher flow rates than typically prescribed for daily use.^25^ High flow rates require heavier devices that are inconvenient to carry and may even impair performance due to the added weight.^27^

Thus, while high-dose SOT can acutely improve exercise performance,^18–20 25^ its effectiveness in real-life settings, where low-flow ambulatory and often pulsed devices are commonly prescribed, remains unclear. Factors such as delivery mode (mask vs cannula), mouth breathing, device weight, flow rate and triggers may all influence outcomes. Because ambulatory SOT is commonly prescribed despite these limitations, it is clinically relevant to determine whether walking without low-dose SOT results in clinically meaningful reductions in exercise performance. Demonstrating that exercise capacity in ambient air is not clinically worse than with commonly prescribed low-flow SOT addresses a relevant and unresolved question in patient management. Therefore, we adopted a non-inferiority framework to directly address whether patients can maintain functional walking performance without routine use of low-dose ambulatory SOT.

The research question was whether ambient air was non-inferior to low-flow SOT via portable oxygen concentrators, as used in everyday life by patients with COPD or ILD and exercise-induced desaturation, in terms of improving 6MWD.

## Methods

### Study design

This study was designed as a randomised, controlled, non-inferiority crossover trial. Blinding was omitted, as the design aimed to mimic real-life application. The trial was part of the broader 6MWT desaturators project at the University Hospital of Zurich, Switzerland, and followed the same study protocol as the companion study performed in patients with pulmonary arterial hypertension and chronic thromboembolic hypertension.^28^ The project was registered at ClinicalTrials.gov (NCT06385301). Recruitment and measurements took place from May 2024 to July 2025.

### Study population

Eligible patients were adult women and men with a diagnosis of COPD or ILD who demonstrated an oxygen desaturation of >3% on pulse oximetry during their most recent standardised exercise test at our centre, typically a 6MWT performed as part of their routine follow-up. Excluded were patients with inability to follow the study protocol due to language barriers, psychological, neurological or orthopaedic conditions limiting walking ability, pregnancy or severe resting hypoxaemia (PaO_2_ <6.9 kPa). We recruited patients during routine ambulatory visits for COPD or ILD at the University Hospital Zurich, Switzerland. Those who met the eligibility criteria were contacted by phone, informed about the study and its requirements and invited to participate. During their subsequent clinic visit, the inclusion and exclusion criteria were reassessed.

### Study procedure, assessments and outcomes

Each patient completed two 6MWT, one in ambient air and one with SOT, following current guidelines.^29^ The tests were conducted in randomised order, with a minimum wash-out period of at least 1 hour between them. An investigator independent of the measurements generated and stored the allocation sequence. Enrolling investigators assigned patients consecutively to pregenerated study IDs to maintain allocation concealment. Participants who were unfamiliar with SOT devices were instructed on their use (eg, to breathe through the nose whenever possible). Nasal breathing during the test was not controlled as effectiveness was evaluated not efficacy. The recruited participants were already familiar with 6MWTs from clinical use; therefore, no familiarisation 6MWTs were performed. Participants already under low-dose long-term SOT performed the ambient air test without oxygen.

For the 6MWT with SOT, a portable concentrator (Philips SimplyGo Mini, weight 2.3 kg) carried in a backpack, as is common in clinical practice, was used. The device was set to the maximum level (level 5), which delivered a 50 mL bolus of oxygen triggered per breath. At a breathing rate of 20 breaths per minute, this results in approximately 1 L/min, which increases with higher respiratory frequencies during exercise. SOT was initiated 5 min before baseline assessments and maintained throughout the test, including the measurements at end-exercise. The 6MWT without intervention was performed without additional weight (eg, a backpack simulating the concentrator’s weight) to reflect real-life conditions.

The primary endpoint was the mean difference in 6MWD of ambient air minus SOT. Secondary endpoints included SpO_2_, blood pressure and heart rate, all measured at baseline and immediately at the end of each test, as well as Borg CR10 scores for dyspnoea and leg fatigue at end-exercise.^30^ After the second 6MWT, patients were also asked to indicate their preferred condition and provide reasons for their choice. For baseline parameters such as arterial blood gas analysis and lung function, the most recent test performed in the clinic was used.

### Statistical analysis and sample size

For the sample size calculation, a non-inferiority margin of −35 m for the primary endpoint 6MWD was used, as it represents the minimal clinically important difference (MCID) for COPD.^31 32^ In ILD, the MCID for the 6MWT is less established and ranges, depending on methods, from 21.7 to 37 m.^33^ Because of this wide range, it was decided to use −35 m for both cohorts, keeping in mind that it might not reflect the ILD cohort perfectly. Based on the method outlined by Rothmann *et al*^34^ and assuming a within-subject SD of 45 m,^32 35^ α=0.05, β=0.2, and an expected difference of −8 m, a sample size of 18 patients was required. To account for potential uncertainties, the target was increased to 20 patients per group (COPD or ILD), resulting in a total of 40 patients.

Randomisation to the two conditions (SOT and ambient air) was performed using block randomisation with randomly permuted blocks of four. Descriptive statistics are presented as means with SD. The primary outcome, 6MWD, was analysed using a linear mixed-effects model in an intention-to-treat (ITT) framework. Missing data were handled implicitly by the model under a missing-at-random assumption. Non-inferiority was assessed against the predefined margin of –35 m. To check for robustness of results, a sensitivity analysis between ITT and per protocol (PP) was performed. Model assumptions, including homogeneity and normality of residuals, were evaluated visually using Tukey-Anscombe and Q-Q plots. To enhance model precision, potential covariates, including diagnosis, baseline diffusing capacity of the lungs for carbon monoxide (DLCO), sex, age and body mass index (BMI), as well as period and carry-over effects, were examined. In the exploratory analyses, we also assessed whether baseline characteristics were associated with clinically meaningful improvements in 6MWD. Responders were defined as participants achieving an increase of >35 m (clinically significant responders) or >20 m (exploratory responders). The lower, exploratory threshold was introduced to account for a potential ceiling effect, as participants who already walked long distances in ambient air may have had limited scope for further improvement and therefore might not have reached the established MCID. Secondary outcomes were analysed using the same statistical method but tested with two-sided hypotheses. Missing data in the secondary outcomes were not replaced. All analyses were conducted in RStudio (V.4.5.0), with statistical significance set at p<0.05.

## Results

### Baseline characteristics

**Figure 1:**
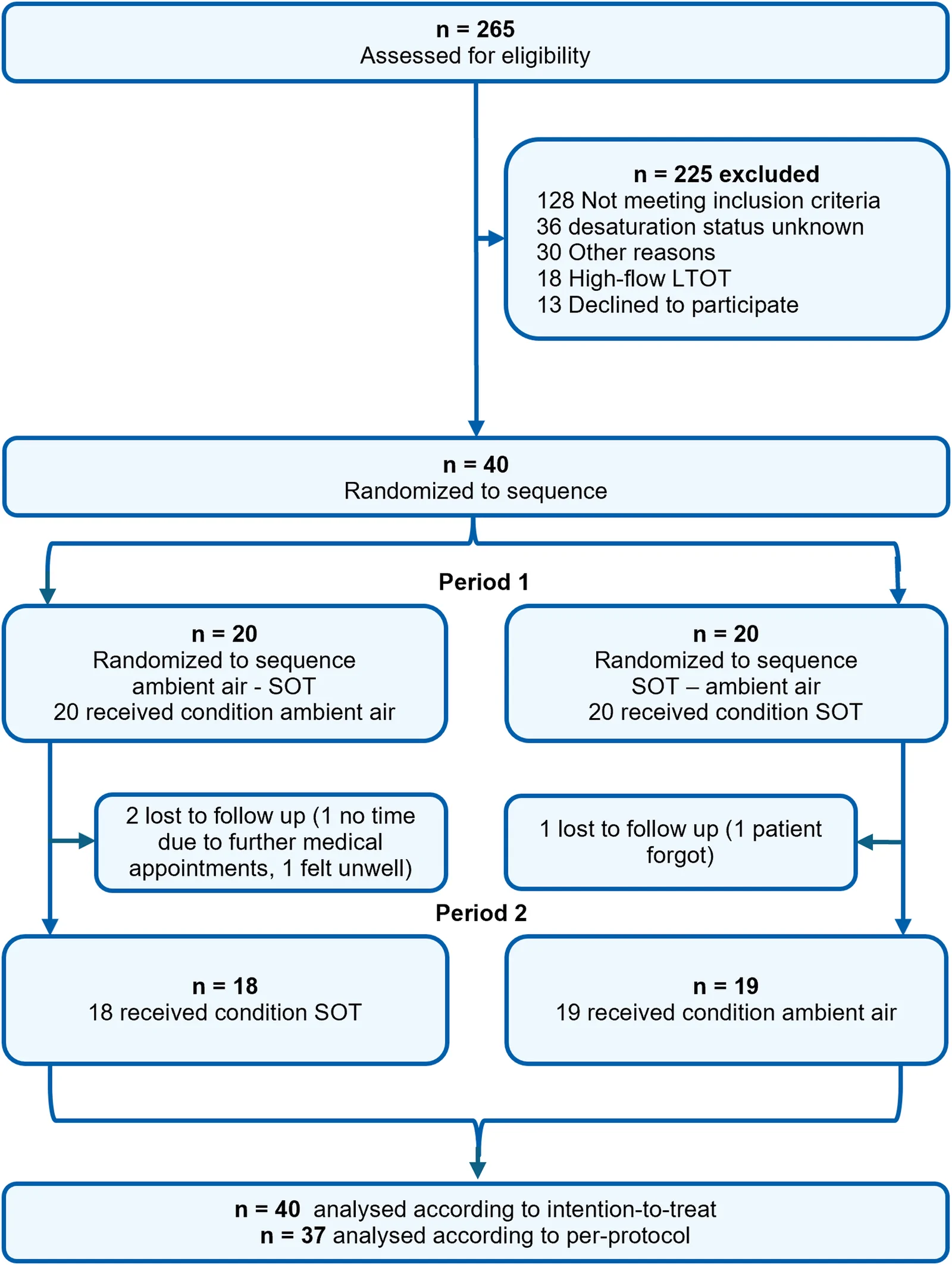
Consolidated Standards of Reporting Trials (CONSORT) flow chart for crossover studies. LTOT, long-term oxygen therapy; SOT, supplemental oxygen therapy.

Figure 1 illustrates the patient flow. Three patients did not complete the second measurement: one forgot (ILD), one had to undergo further medical assessments (COPD) and one dropped out due to general discomfort (ILD). Overall, there were more men (n=26) than women (n=14) included. Age, BMI and New York Heart Association class were similarly distributed between groups and were representative of the respective populations. Baseline characteristics are summarised in table 1.

**Table 1:** Baseline characteristics of the COPD and ILD group separately and combined

Baseline characteristics	All	COPD	ILD
N	40	20	20
Male	26 (65%)	14 (70%)	12 (60%)
Female	14 (35%)	6 (30%)	8 (40%)
Age (years)	66±13	69±8	64±16
BMI (kg/m^2^)	25.8±5.0	26.6±4.9	24.9±5.0
NYHA class			
1–2	31 (77.5%)	17 (85%)	14 (70%)
3–4	8 (20%)	3 (15%)	5 (25%)
NA	1 (2.5%)	0	1 (5%)
DLCO (% predicted)	47.7±20.5	46.9±19.4	48.6±22.2
KCO (% predicted)	67.1±21.5	59.7±21.3	74.5±19.4
FVC (% predicted)	74.9±26.7	73.9±26.8	76.0±27.3
FEV_1_ (% predicted)	67±25	57±25	76±21
FEV_1_/FVC	69.4±16.6	58.1±14.6	81.4±8.1
TLC (% predicted)	84±25	99±16	69±22
RV/TLC	39.2±8.5	43.3±9.2	34.9±5.2
pH	7.42±0.04	7.43±0.04	7.42±0.05
PaO_2_ (kPa)	9.7±1.8	9.2±1.9	10.2±1.6
PaCO_2_ (kPa)	4.9±0.6	5.0±0.8	4.9±0.5
SpO_2_ aBGA (%)	94±3	93±4	95±2
Hb (g/L)	134±42	152±12	117±52
Lactate (mmol/L)	1.14±0.84	0.89±0.43	1.37±1.05
Low-flow LTOT	3 (7.5%)	0 (0%)	3 (15%)

Data are given as mean±SD unless indicated otherwise.

aBGA, arterial blood gas analysis; BMI, body mass index; COPD, chronic obstructive pulmonary disease; DLCO, diffusing capacity of the lungs for carbon monoxide; FEV_1_, forced expiratory volume 1 s; FVC, forced ventilatory capacity; Hb, haemoglobin; ILD, interstitial lung disease; KCO, carbon monoxide transfer coefficient; LTOT, long-term oxygen therapy; NA, not available; NYHA, New York Heart Association; PaCO_2_, partial pressure of carbon monoxide in the arterial blood; PaO_2_, partial pressure of oxygen in the arterial blood; RV, reserve volume; SpO_2_, oxygen saturation; TLC, total lung capacity.

### 6-Minute walk distance

Ambient air was non-inferior to SOT for the 6MWD, with a mean difference of 0 m (95% CI –14 to 13 m; p<0.001), with consistent results in the COPD and ILD subgroups (table 2). Sensitivity analyses in the ITT and PP populations confirmed the robustness of the findings (online supplemental figure 1). None of the examined covariates (diagnosis, sex, age, predicted DLCO, BMI) were significant, and no period or carry-over effects were observed. The final model therefore included only participant ID as a random effect.

**Table 2:** 6MWD outcomes combined and separate per diagnosis group

6MWD (m)	Ambient air Mean±SD	SOT Mean±SD	Paired mean difference (95% CI) Air-SOT	One-sided p value
All CLD	463±111	464±111	0 (−14 to 13)	**<0.001**
COPD	474±84	468±84	1 (−14 to 17)	**<0.001**
ILD	452±136	458±136	−2 (−27 to 23)	**0.006**

For the means and differences, the true values were used. 95% CIs and p values were calculated using the model. Differences between conditions were calculated as ambient air minus oxygen. Significant p values are marked bold.

CLD, chronic lung disease; CPOD, chronic obstructive pulmonary disease; ILD, interstitial lung disease; 6MWD, 6-minute walk distance; SOT, supplemental oxygen therapy.

### Baseline characteristics as predictors of response

We observed five patients who met the threshold for a clinically meaningful response (>35 m improvement in 6MWD) and three patients who fell within the exploratory responder range (20–35 m) with SOT. Descriptive exploration indicated that both clinically meaningful and exploratory responders tended to have lower baseline 6MWD values in ambient air, with the clinically meaningful responder group exhibiting the shortest distances (see online supplemental table 1). In contrast, oxygen saturation at rest and at end-exercise predicted DLCO and WHO functional class were not associated with response to SOT.

In an exploratory Firth logistic regression analysis, a shorter baseline 6MWD in ambient air was significantly linked to higher odds of responding to SOT (OR 0.989, 95%CI 0.976 to 0.999; p=0.023). This corresponds to a 1.1% reduction in the likelihood of response for every additional metre walked at baseline.

### Secondary outcomes

Dyspnoea scores were significantly lower with SOT in the whole CLD group (1, 95% CI 1 to 2; p<0.001) as well as in COPD (1, 95% CI 0.05 to 2; p=0.041) and in ILD (2, 95% CI 1 to 3; p=0.005). There was no evidence for a difference between conditions for any of the other secondary outcomes, either overall or within subgroups (table 3).

**Table 3:** Secondary outcomes combined and separate per diagnosis group

Overall chronic lung diseases								
**Outcome**	**Ambient air**			**SOT**			**Mean difference ambient air—SOT (95% CI)**	**Two-sided p values**
	Rest	End-exercise	Paired difference (end-rest)	Rest	End-exercise	Paired difference (end-rest)		
Dyspnoea (Borg)	NA	5±3	NA	NA	4±3	NA	1 (1 to 2)	**<0.001**
Leg fatigue (Borg)	NA	2±3	NA	NA	2±3	NA	0 (0 to 1)	0.482
SpO_2_ (%)	94±4	86±8	−9±6	96±3	88±7	−8±7	−1 (−2 to 1)	0.562
HR (bpm)	87±17	112±18	25±14	84±14	110±18	26±14	−1 (−3 to 3)	0.887
Systolic BP (mm Hg)	131±19	153±27	21±20	132±18	152±28	19±21	2 (−5 to 10)	0.503
Diastolic BP (mm Hg)	85±14	93±16	7±14	86±15	90±11	4±13	3 (−3 to 10)	0.251
**Chronic obstructive pulmonary disease**								
Dyspnoea (Borg)	NA	5±3	NA	NA	4±3	NA	1 (0 to 2)	**0.041**
Leg fatigue (Borg)	NA	3±3	NA	NA	2±2	NA	1 (0 to 1)	0.144
SpO_2_ (%)	94±3	86±7	−8±5	95±3	87±7	−8±7	0 (−3 to 2)	0.608
HR (bpm)	88±14	112±17	24±13	87±15	110±17	21±11	3 (−3 to 4)	0.869
Systolic BP (mm Hg)	132±16	156±28	24±24	134±16	155±26	21±22	3 (−10 to 16)	0.669
Diastolic BP (mm Hg)	85±11	95±16	10±16	85±11	89±12	4±10	6 (−3 to 14)	0.200
**Interstitial lung disease**								
Dyspnoea (Borg)	NA	5±3	NA	NA	4±3	NA	1 (1 to 3)	**0.005**
Leg fatigue (Borg)	NA	2±3	NA	NA	2±3	NA	0 (−1 to 1)	0.823
SpO_2_ (%)	95±4	86±8	−9±7	97±2	88±8	−9±7	0 (−3 to 2)	0.816
HR (bpm)	86±20	112±19	26±16	82±12	110±18	28±17	−2 (−6 to 4)	0.757
Systolic BP (mm Hg)	130±22	149±24	18±13	130±20	148±31	17±19	1 (−5 to 10)	0.446
Diastolic BP (mm Hg)	84±18	90±16	4±9	88±18	91±13	3±18	1 (−7 to 9)	0.879

For the remaining variables, end-exercise means and differences were calculated from the true values. 95% CIs and p values were calculated using the model. Differences within conditions were calculated as end-exercise minus rest. Differences between conditions were calculated as ambient air minus SOT. Significant p values are marked bold.

BP, blood pressure; HR, heart rate; NA, not available; SOT, supplemental oxygen therapy; SpO_2_, oxygen saturation.

### Subjective preference

**Figure 2:**
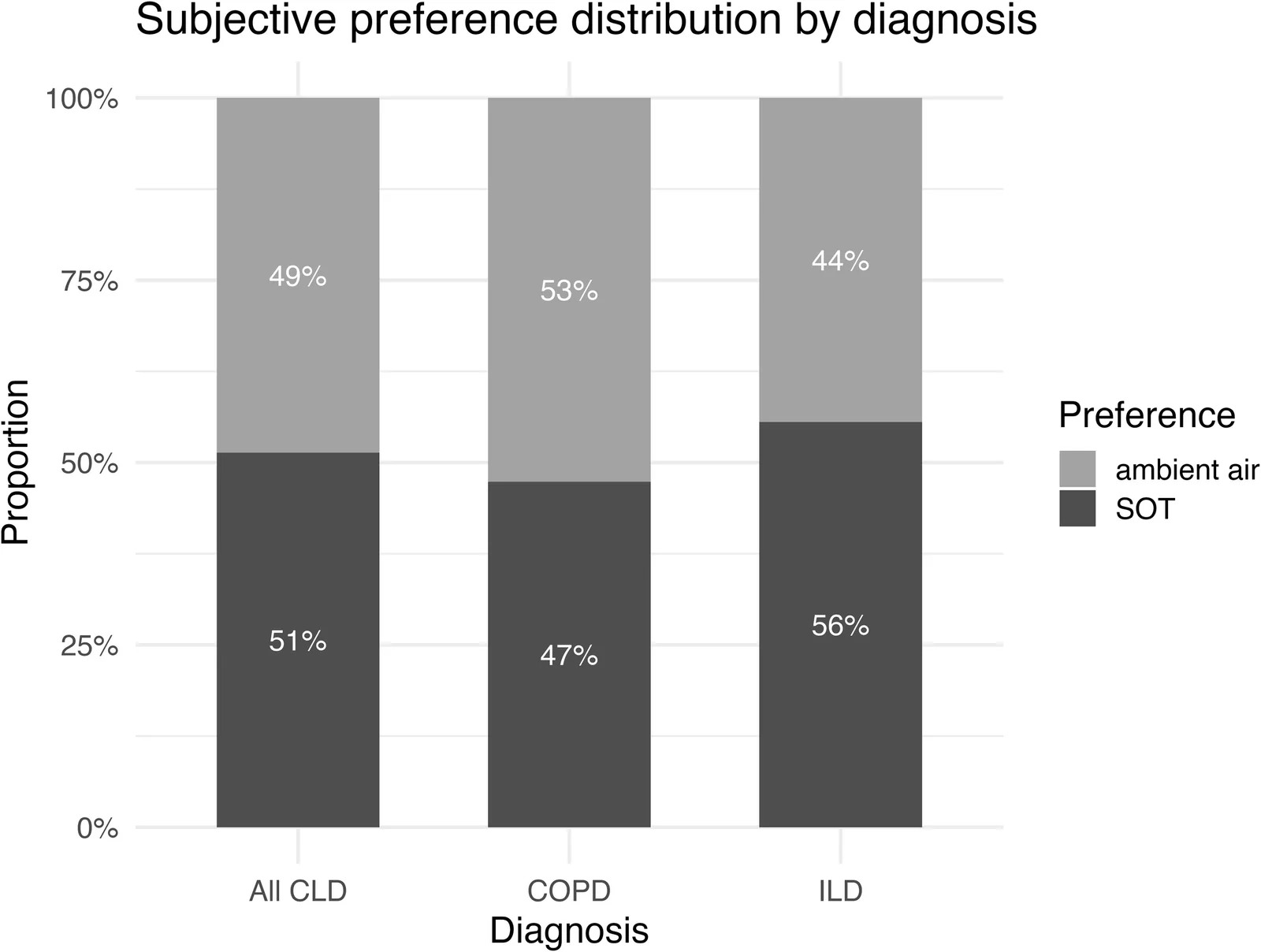
Visualisation of the preference by diagnosis. CLD, chronic lung disease; COPD, chronic obstructive pulmonary disease; ILD, interstitial lung disease.

Overall, 49% of patients preferred ambient air and 51% favoured SOT (figure 2). When analysed by diagnosis, among patients with COPD 53% preferred ambient air and 47% preferred SOT. In the ILD group, 44% favoured ambient air and 56% preferred SOT. Preference was not associated with better performance in the preferred condition.

During post-test interviews, patients who favoured ambient air frequently reported perceiving no subjective benefit from SOT and described the device and nasal cannula as bothersome. For instance, one patient stated to have “*no subjective benefit*” (ID 231), while another remarked the “*smell of plastic, weight and vibration of backpack are disturbing*” (ID 215). Similarly, a third participant commented, “*I felt no benefit from oxygen. I breathe through the mouth when under load, and therefore I think SOT did not help because of that*” (ID 240).

In contrast, patients who preferred SOT often highlighted reductions in dyspnoea and fatigue, and several thought that SOT allowed them to walk longer distances. One participant described, “*I had less coughing and dyspnea with oxygen and feel that I could go further with oxygen*” (ID 232), and another stated “*I could walk faster with oxygen*” (ID 208). Additionally, two patients mentioned that they feel they would benefit more with higher oxygen dosages (ID 213, 219).

## Discussion

In this pragmatic randomised controlled trial (RCT reflecting real-world conditions, we showed for the first time that in patients with CLD and exercise-induced oxygen desaturation, walking in ambient air was non-inferior to pulsed low-dose SOT administered via nasal cannula from a commonly prescribed, portable backpack concentrator. This was most likely due to the relatively low oxygen dose of around 1 L/min received via nasal cannula during ambulation and the additional weight of the concentrator. In contrast, walking with SOT was linked to lower perceived dyspnoea, and reflecting this subjective advantage, half of the patients expressed a preference for SOT over ambient air with no relevant between-group differences.

The most recent meta-analysis in patients with advanced progressive illness, mainly COPD or ILD, reported a significant improvement in 6MWD with SOT compared with medical air (standardised mean difference of 0.23, 95% CI 0.10 to 0.36).^36^ However, these findings are not directly comparable to ours, as several included studies used higher oxygen flow rate (3–5 L/min) or oronasal masks instead of nasal cannulas as used in our study reflecting a real-life setting. Ambient air meeting the non-inferiority criteria is most likely explained by inadequate oxygen delivery during walking, caused by dilution of low-flow nasal oxygen through mouth breathing, lack of triggered activation of the concentrator and the additional burden of carrying the portable concentrator, which may have offset any potential benefits of SOT. While higher SOT flow systems or sealed interfaces could improve alveolar oxygenation, their size, weight and limited acceptance restrict real-world use. Moreover, the above-mentioned meta-analysis did not evaluate whether the observed effect was clinically meaningful. Assuming an SD of 120 m, as reported in a large COPD cohort,^37^ the pooled standardised mean difference translates to an average 6MWD gain of about 28 m (12–43 m), which is below the MCID.^32^ To date, no other non-inferiority trial has directly investigated SOT vs ambient air in this population.

Despite ambient air being non-inferior, most patients in our study reported lower perceived dyspnoea when walking with SOT, consistent with prior findings both in COPD and ILD.^24 36 38^ This suggests that SOT may provide symptomatic relief by reducing the sensation of breathlessness, which could in turn influence walking endurance beyond a 6-min walking period.

However, this symptomatic benefit must be weighed against the practical drawbacks reported by patients, particularly among the COPD group, who described SOT as physically burdensome due to the device’s weight, warmth and vibration. The nasal cannula was also perceived as uncomfortable, with some patients noting that they do not breathe through their nose, especially during exertion. Similar observations have been reported in a qualitative study of patients with ILD, caregivers and healthcare professionals, emphasising the need for lighter, more user-friendly yet more powerful oxygen delivery systems.^39^ Notably, up to 50% of patients preferred walking in ambient air because they did not experience a meaningful subjective benefit from SOT.

### Responder versus non-responder

The exploratory descriptive analysis indicated that younger and more severely ill patients were more likely to be classified as clinically significant responders. In the logistic regression model, the only variable associated with response was the baseline 6MWD in ambient air. Given the very small number of responders (n=5) and the resulting wide CIs, the estimates are highly unstable and the degree of uncertainty should be considered substantial.

A similar finding regarding baseline 6MWD as a predictor of response was reported in an RCT on COPD and short-term low-flow SOT, which showed that responders to SOT had significantly lower 6MWD in ambient air.^38^ In that study, 42% of the patients with exercise-induced desaturation improved by >30 m with SOT (2 L/min),^38^ whereas in our study, only 14% were classified as clinically significant responders. Even with lowering the cut-off to 30 m for the MCID, there would have been no additional responders. Importantly, the mean 6MWD in ambient air was considerably lower in the mentioned trial (377±96 m) compared with the present study (463±111 m).

The apparent predictive value of baseline 6MWD, as well as the discrepancy between the two studies, may be explained by the known ceiling effect of the 6MWT. Prior work in COPD has suggested that this ceiling effect occurs at 6MWD >450–500 m, as correlation with peak VO_2_ weakens beyond this range.^40^ This phenomenon has also been supported by studies in pulmonary hypertension.^41–43^ Patients who have already achieved high 6MWD at baseline have limited potential for further improvement, which is consistent with the lower response rates observed in our study. However, the association between baseline capacity and oxygen response has also been demonstrated using exercise tests without a pronounced ceiling effect. For example, in idiopathic pulmonary fibrosis, patients with shorter endurance times in placebo air improved more with SOT in a constant work-rate exercise test.^24^ This observation may reflect that individuals with more restricted exercise capacity due to ventilatory limitation or hypoxaemia derive greater benefit from oxygen therapy.

Further studies using exercise tests without inherent ceiling limitations are needed to clarify whether lower baseline performance in ambient air truly predicts a greater likelihood of response to SOT. This could help identify patients who would benefit most from SOT.

### Differential response of COPD versus ILD

The ILD group comprised a broad spectrum of diagnoses, reflecting the heterogeneous character of this disease category. In total, 11 distinct ILD diagnoses were represented, with additional patients classified as having ILD of unknown aetiology. Three patients required long-term SOT at flow rates of 1.5–2.0 L/min, indicating that varying degrees of disease severity were included. This diversity likely contributed to the wide variability in the 6MWD response to SOT observed within the ILD group. However, the chosen crossover RCT with patients being their own controls allows firm conclusions despite a certain heterogeneity. In contrast, the COPD cohort was more homogeneous, as reflected by the narrower CIs for 6MWD.

Besides the already mentioned slightly larger dyspnoea improvement of the ILD group compared with COPD, no other relevant between-group differences were observed. A reason for this could be the difference in ventilatory drive or the difference in pathophysiology.

### Limitations

This study was deliberately conducted without sham control, as we wanted to consider the weight of the concentrator versus the potential benefits of SOT according to real-life use, and therefore, blinding was not intended. Nevertheless, both the additional weight of the concentrator and patients’ awareness of receiving either SOT or ambient air may have introduced bias. This is particularly important to consider for subjective outcomes such as dyspnoea, leg fatigue and treatment preference, but also for the objective 6MWD, given potential differences in exertional efforts. Exercise-induced desaturation was defined as a decrease in SpO_2_ of >3% rather than by a nadir threshold of <88%, which may have resulted in the inclusion of patients with less severe desaturation. Additionally, SpO_2_ was assessed at rest and immediately at completion of the 6MWT rather than by continuous oximetry. Therefore, saturation nadir and time spent below clinically relevant thresholds (eg, SpO_2_ ≤88%) could not be determined, and the present study cannot establish whether low-flow SOT reduced the depth or duration of exertional desaturation. Furthermore, the short exposure to SOT during the 6MWT does not allow for conclusions regarding long-term use, such as nocturnal or domiciliary oxygen therapy and their benefits.^44 45^

## Conclusion

This pragmatic RCT showed that ambient air was non-inferior to pulsed low-dose SOT delivered via a portable concentrator via nasal cannula to improve 6MWD in patients with CLD and exercise-induced desaturation. However, the absence of a clinically meaningful improvement in 6MWD should not be interpreted as an absence of clinical benefit as SOT reduced dyspnoea in both COPD and ILD. Baseline 6MWD appears to influence responsiveness to SOT, with lower distances in ambient air being associated with a greater likelihood of improvement. These findings should be further investigated using exercise tests without a ceiling effect and efforts should focus on developing lighter, more patient-acceptable SOT delivery systems.

## Supplementary material

10.1136/bmjresp-2025-004048 online supplemental file 1

## Data Availability

Data are available on reasonable request.

## References

[1] Khor YH, Gutman L, Abu Hussein N (2021). Incidence and Prognostic Significance of Hypoxemia in Fibrotic Interstitial Lung Disease: An International Cohort Study. Chest.

[2] Khor YH, Goh NS, Glaspole I (2019). Exertional Desaturation and Prescription of Ambulatory Oxygen Therapy in Interstitial Lung Disease. Respir Care.

[3] van Gestel AJR, Clarenbach CF, Stöwhas AC (2012). Prevalence and prediction of exercise-induced oxygen desaturation in patients with chronic obstructive pulmonary disease. Respiration.

[4] Dogra AC, Gupta U, Sarkar M (2015). Exercise-induced desaturation in patients with chronic obstructive pulmonary disease on six-minute walk test. Lung India.

[5] Kim C, Ko Y, Lee JS (2021). Predicting long-term mortality with two different criteria of exercise-induced desaturation in COPD. Respir Med.

[6] Emmanuel GE, Moreno F (1966). Distribution of ventilation and blood flow during exercise in emphysema. J Appl Physiol.

[7] Cutillo A, Bigler AH, Perondi R (1981). Exercise and distribution of inspired gas in patients with obstructive pulmonary disease. Bull Eur Physiopathol Respir.

[8] Zafar MA, Tsuang W, Lach L (2013). Dynamic hyperinflation correlates with exertional oxygen desaturation in patients with chronic obstructive pulmonary disease. Lung.

[9] Ahmed MS, Neyaz A, Aslami AN (2016). Health-related quality of life of chronic obstructive pulmonary disease patients: Results from a community based cross-sectional study in Aligarh, Uttar Pradesh, India. Lung India.

[10] Miravitlles M, Ribera A (2017). Understanding the impact of symptoms on the burden of COPD. Respir Res.

[11] Watz H, Pitta F, Rochester CL (2014). An official European Respiratory Society statement on physical activity in COPD. Eur Respir J.

[12] Garcia-Aymerich J, Lange P, Benet M (2006). Regular physical activity reduces hospital admission and mortality in chronic obstructive pulmonary disease: a population based cohort study. Thorax.

[13] Yohannes AM, Baldwin RC, Connolly M (2002). Mortality predictors in disabling chronic obstructive pulmonary disease in old age. Age Ageing.

[14] Vaes AW, Garcia-Aymerich J, Marott JL (2014). Changes in physical activity and all-cause mortality in COPD. Eur Respir J.

[15] Flaherty KR, Andrei A-C, Murray S (2006). Idiopathic pulmonary fibrosis: prognostic value of changes in physiology and six-minute-walk test. Am J Respir Crit Care Med.

[16] Haidl P, Jany B, Geiseler J (2020). Guideline for Long-Term Oxygen Therapy - S2k-Guideline Published by the German Respiratory Society. Pneumologie.

[17] Russi EW, Karrer W, Brutsche M (2013). Diagnosis and Management of Chronic Obstructive Pulmonary Disease: The Swiss Guidelines. *Respiration*.

[18] Ulrich S, Hasler ED, Saxer S (2017). Effect of breathing oxygen-enriched air on exercise performance in patients with precapillary pulmonary hypertension: randomized, sham-controlled cross-over trial. Eur Heart J.

[19] Ulrich S, Hasler ED, Furian M (2017). Mechanisms of Improved Exercise Performance under Hyperoxia: On Haldane, Geppert, Zunz, and Eschenbacher Transformations. Respiration.

[20] Hasler ED, Saxer S, Schneider SR (2020). Effect of Breathing Oxygen-Enriched Air on Exercise Performance in Patients with Chronic Obstructive Pulmonary Disease: Randomized, Placebo-Controlled, Cross-Over Trial. Respiration.

[21] van Helvoort HAC, Heijdra YF, Heunks LMA (2006). Supplemental oxygen prevents exercise-induced oxidative stress in muscle-wasted patients with chronic obstructive pulmonary disease. Am J Respir Crit Care Med.

[22] Alison JA, McKeough ZJ, Leung RWM (2019). Oxygen compared to air during exercise training in COPD with exercise-induced desaturation. Eur Respir J.

[23] Wadell K, Henriksson-Larsén K, Lundgren R (2001). Physical training with and without oxygen in patients with chronic obstructive pulmonary disease and exercise-induced hypoxaemia. J Rehabil Med.

[24] Arizono S, Furukawa T, Taniguchi H (2020). Supplemental oxygen improves exercise capacity in IPF patients with exertional desaturation. Respirology.

[25] Archontakis Barakakis P, Wolfe A, Schwartz A (2024). Supplementary oxygen efficacy for chronic pulmonary disorders and exertion desaturation. ERJ Open Res.

[26] Bell EC, Cox NS, Goh N (2017). Oxygen therapy for interstitial lung disease: a systematic review. Eur Respir Rev.

[27] Ramadurai D, Riordan M, Graney B (2018). The impact of carrying supplemental oxygen on exercise capacity and dyspnea in patients with interstitial lung disease. Respir Med.

[28] Wick C, Lichtblau M, Titz A (2026). Effect of nasal low-flow oxygen therapy vs. ambient air on 6-minute walk distance in patients with pulmonary vascular disease revealing exercise-induced desaturation. A pragmatic randomized-controlled non-inferiority cross-over trial., Forthcoming.

[29] ACoPSfCPF L (2002). ATS Statement: Guidelines for the Six-Minute Walk Test. Am J Respir Crit Care Med.

[30] Borg GAV (1982). Psychophysical bases of perceived exertion. Medicine & Science in Sports & Exercise.

[31] Puhan MA, Mador MJ, Held U (2008). Interpretation of treatment changes in 6-minute walk distance in patients with COPD. Eur Respir J.

[32] Mathai SC, Puhan MA, Lam D (2012). The minimal important difference in the 6-minute walk test for patients with pulmonary arterial hypertension. Am J Respir Crit Care Med.

[33] Nathan SD, du Bois RM, Albera C (2015). Validation of test performance characteristics and minimal clinically important difference of the 6-minute walk test in patients with idiopathic pulmonary fibrosis. Respir Med.

[34] Rothmann MD, Wiens BL, Chan ISF (2020). Design and analysis of non-inferiority trials.

[35] du Bois RM, Weycker D, Albera C (2011). Six-minute-walk test in idiopathic pulmonary fibrosis: test validation and minimal clinically important difference. Am J Respir Crit Care Med.

[36] Hasegawa T, Ochi T, Goya S (2023). Efficacy of supplemental oxygen for dyspnea relief in patients with advanced progressive illness: A systematic review and meta-analysis. Respir Investig.

[37] Celli B, Tetzlaff K, Criner G (2016). The 6-Minute-Walk Distance Test as a Chronic Obstructive Pulmonary Disease Stratification Tool. Insights from the COPD Biomarker Qualification Consortium. Am J Respir Crit Care Med.

[38] Jarosch I, Gloeckl R, Damm E (2017). Short-term Effects of Supplemental Oxygen on 6-Min Walk Test Outcomes in Patients With COPD: A Randomized, Placebo-Controlled, Single-blind, Crossover Trial. Chest.

[39] Tikellis G, Hoffman M, Mellerick C (2023). Barriers to and facilitators of the use of oxygen therapy in people living with an interstitial lung disease: a systematic review of qualitative evidence. Eur Respir Rev.

[40] Chae G, Ko EJ, Lee SW (2022). Stronger correlation of peak oxygen uptake with distance of incremental shuttle walk test than 6-min walk test in patients with COPD: a systematic review and meta-analysis. BMC Pulm Med.

[41] Frost AE, Langleben D, Oudiz R (2005). The 6-min walk test (6MW) as an efficacy endpoint in pulmonary arterial hypertension clinical trials: demonstration of a ceiling effect. Vascul Pharmacol.

[42] Lachant D, Kennedy E, Derenze B (2022). Cardiac Effort to Compare Clinic and Remote 6-Minute Walk Testing in Pulmonary Arterial Hypertension. Chest.

[43] Appenzeller P, Gautschi F, Müller J (2022). Prediction of maximal oxygen uptake from 6-min walk test in pulmonary hypertension. ERJ Open Res.

[44] Khor YH, Palm A, Wong AW (2025). Effects of long-term oxygen therapy on acute exacerbation and hospital burden: the national DISCOVERY study. Thorax.

[45] Ekström M, Andersson A, Papadopoulos S (2024). Long-Term Oxygen Therapy for 24 or 15 Hours per Day in Severe Hypoxemia. N Engl J Med.

