# Supplementary material for: Portable low-flow nasal oxygen versus ambient air in patients with chronic lung disease and exercise-induced hypoxaemia: a randomised controlled crossover non-inferiority trial
Source: BMJ Open Respir Res. 2026 Sep 21;13(1):e004048. doi: 10.1136/bmjresp-2025-004048 (PMC13599829; doi:10.1136/bmjresp-2025-004048)
Supplement: Supplementary file 1 [file bmjresp-13-1-s001.docx]

**Supplementary Material**

**Responder vs. non-responder characteristics**

|  | **Clinically significant Responders (**>**35 m)** | **Exploratory responder (20-35 m)** | **Non-Responder** |
| --- | --- | --- | --- |
| n | 5 | 3 | 28 |
| Male | 3 (60 %) | 1 (33 %) | 19 (68 %) |
| Female | 2 (40 %) | 2 (67 %) | 9 (32 %) |
| Age (years) | 59 ± 11 | 67 ± 4 | 68 ± 11 |
| BMI (kg/m^2^) | 30.4 ± 7.6 | 23.6 ± 2.4 | 25.8 ± 4.3 |
| NYHA Class |  |  |  |
| 1-2 | 3 (60 %) | 2 (67 %) | 24 (86 %) |
| 3-4 | 2 (40 %) | 1 (33 %) | 4 (14 %) |
| Diagnosis |  |  |  |
| COPD | 3 (60 %) | 0 (0 %) | 16 (52 %) |
| ILD | 2 (40 %) | 3 (100 %) | 15 (48 %) |
| 6MWD (m) |  |  |  |
| Ambient air | 378 ± 91 | 454 ± 88 | 493 ± 93 |
| SOT | 447 ± 48 | 475 ± 89 | 479 ± 99 |
| DLCO(% predicted) | 47 ± 20 | 28 ± 6 | 48 ± 21 |
| Predicted FEV_1_ (%) | 47 ± 10 | 79 ± 19 | 67 ± 26 |
| Predicted TLC (%) | 70 ± 36 | 53 ± 10 | 89 ± 24 |
| pH | 7.44 ± 0.05 | 7.45 ± 0.01 | 7.42 ± 0.04 |
| PaO_2_ (kPa) | 9.32 ± 1.69 | 8.93 ± 0.53 | 9.56 ± 1.79 |
| PaCO_2_ (kPa) | 5.11 ± 0.29 | 4.83 ± 0.29 | 4.96 ± 0.75 |
| SpO_2_ (%) | 94 ± 3 | 95 ± 1 | 93 ± 4 |
| Hb (g/l) | 127 ± 65 | 136 ± 17 | 136 ± 43 |
| Lactate (mmol/l) | 0.92 ± 0.40 | 1.63 ± 1.79 | 1.06 ± 0.70 |
| Low-flow LTOT | 1 (20 %) | 0 (0 %) | 2 (7 %) |

Supplemental Table 1: Characteristics of responder vs. non-responders. Only patients with completion of both 6MWT were included. Abbreviations: Body mass index (BMI), diffusing capacity of the lungs for carbon monoxide (DLCO), partial pressure of oxygen in the arterial blood (PaO2), partial pressure of carbon monoxide in the arterial blood (PaCO2), hemoglobin (Hb), oxygen saturation (SpO2).

**Sensitivity analysis**

Supplemental Figure 1: Sensitivity Analysis by diagnoses comparing per protocol (PP) and intention to treat (ITT) analysis. Abbreviations: Chronic lung disease (CLD), chronic obstructive lung disease (COPD), interstitial lung disease (ILD), supplemental oxygen therapy (SOT).
